# Perception of masculinity amongst young Malaysian men: a qualitative study of university students

**DOI:** 10.1186/1471-2458-13-1062

**Published:** 2013-11-11

**Authors:** Zahra Fazli Khalaf, Wah Yun Low, Behzad Ghorbani, Effat Merghati Khoei

**Affiliations:** 1 Medical Education and Research Development Unit, Faculty of Medicine, University of Malaya, Kuala Lumpur 50603, Malaysia; 2 Avicenna Research Institute, ACECR, Tehran, Iran; 3 Iranian National Center of Addiction Studies (INCAS): Risk Behavior Institution, Tehran University of Medical Sciences, Tehran, Iran

**Keywords:** Masculinity, Men’s health, Social construction, Qualitative study, University men

## Abstract

**Background:**

Perception of Masculinity plays an important role in men’s lifestyles and health behaviors. Although, the importance of masculinity has been widely discussed in men’s health literature, very little is known about the meanings of masculinity in the Malaysian setting. This research aimed to explore the meanings of masculinity among Malaysian university men.

**Methods:**

This qualitative study utilized in-depth interviews with 34 young Malaysian university men, aged 20–30 years from three main ethnic groups in Malaysia (Malay, Chinese and Indian). Thematic analysis approach was used to extract data. NVIVO v8 qualitative software was used for data management.

**Results:**

From the data collected several concepts emerged that reflected the meanings of masculinity from the participants’ view points. These meanings were associated with a combination of traditional and non-traditional norms that generally benefit men who behave according to culturally dominant role expectations. These included: “Having a good body shape”, “being respected”, “having success with women”, “being a family man”, and “having financial independence”. Socio-cultural factors, such as family environment, religion, public media and popular life style patterns helped to shape and reinforce the meanings of masculinities among university men.

**Conclusions:**

This study revealed that the university context provided a particular culture for construction and reinforcement of the meanings of masculinities, which should be considered by the educators to help in development of healthy masculinities.

## Background

Construction of gender within the society creates different patterns of expectation for both men and women, which lead to different behaviors and risks [1-3]. Masculinity (manhood) is defined as a set of characteristics, qualities or roles that are generally attributed to men [3,4]. Perception of Masculinity plays an important role in men’s lifestyles and health behaviors [1,5-13]. Although the importance of masculinity in men’s health has been widely discussed in the literature [14-18], little is known about the attributes of masculinity, and men’s perception of the importance of these attributes in their life [19]. According to the traditional masculinity ideology, manliness is reflected through being able to take care of oneself; being tough, strong and healthy; emphasis on competition; devaluation of women; hatred of homosexuals [2,20]; engaging in violent and risk taking behaviors [21,22]; and adopting poor help-seeking behaviors [16-18]. Whereas, according to the ‘gender role strain paradigm’, masculinity is not a fixed entity and there is no single standard for this concept [2,23,24]. In other words, this paradigm considers masculinity ‘ideologies’ rather than masculinity ‘ideology’. Hence, the ideal traits of masculinity are constructed differently for men in different social classes, ethnic groups, regional cultures and life stages [2,24]. Gender norms are powerfully affected by the society and culture, and therefore, socio-cultural changes influence people’s understanding of roles in personal, interpersonal, and social contexts [25].

Studies show that the transformation process is predominant among young people and leads them to create their own sexual subcultures. As a result, young people create discourses about sexuality and gender that often differ from the older traditional norms [26]. While much of the research draws attention to the ways in which globalization and exposure to various communicative networks are central in influencing or transforming the culture of sexuality, it is important to investigate how young people define their sexuality and gender roles [27].

In Western post-industrial societies, young men are still confused about what manhood means to them [28]. For example, in the past, Irish young men were designated the role of protector or defender of their community against foreign invasion, but in the after-peace community these masculine roles have been devalued and criticized as being aggressive or violent behaviors. Such changes in the social and cultural contexts have placed young men in a transitional and paradoxical position without providing them with equipments to cope with the changes [5]. Another study regarding the understanding of masculinity in England showed that young boys were under presure for taking normative gender roles to be recognized as “too much” masculine by not expressing the feelings and not seeking for help, whereas being “not enough” masculine resulted in “isolation and rejection from others, such as peer groups” [29].

Higher educated men may construct the concept of masculinity differently [6,13]. As recently, much scholarly attention has been given to behavioral trends and understanding of masculinity in higher education [4,24]. In a study published by Johns Hopkins University [24], college men equated masculinity with “being confident”, “being respected”, “assuming responsibility” and “embodying prowess”. According to this study, men come to college while having been socialized to take traditional masculinity norms, but college experience with its diversity and exposure to different cultures makes them liberalize and motivates them to change their masculinity ideology [24].

Developing countries such as Malaysia are facing socio-cultural changes. These changes have affected daily life and values of people, especially younger ones, by influencing their perception of gender roles [30,31], male–female interactions [32,33] and risk taking behaviors [34]. An Asian study which explored the important masculinity traits among more than 10,000 men from Malaysia, China, Korea, Japan and Taiwan showed that the perception of masculine traits was different between countries. For instance, having a good job was seen as the most important in Malaysia, but being a family man, having lots of money, being a man of honor, and being in control were the most important masculine traits in Korea, China, Japan and Taiwan respectively. Another study on younger population in Malaysia revealed that the notion of ‘new man’ was popular among young respondents. New man, as opposed to the traditional model, is identified as a slim, nude and groomed man, who takes care of his clothes and his fashion commodities [35].

Since more than 60 percent of Malaysian population is young [36] and the number of students in higher education has increased during the last decade [37], young men are experiencing more intermingling with other cultures and life styles [34] that affect their understanding of masculinity and gender roles. However, the research on masculinity concept is scarce in Malaysia, especially among young men. There was no published data on the understanding of masculinity among young university men in Malaysia prior to this research. In response to this knowledge gap, the present qualitative research aimed to explore the shared masculinity conceptualizations among university men. The main research question that guided this study was: What are the meanings of masculinities among young university men?

This paper focuses on social construction of masculinities, and the term ‘man’ refers to a socially constructed concept rather than the biological sex. Before presenting the findings, we will briefly discuss the theoretical basis of the study and research methodology.

### Theoretical basis of the study

The social construction of masculinities explains that gender and role expectations are related to the environment in which we grow up, as well as how live our lives within a social context. It also emphasizes the influence of social interactions and cultural norms in shaping masculine behaviours and role expectations [2]. In other words, meanings of masculinity are created, modified and put into action by individuals during the process of social interaction. A key assumption here is that masculinity is not a fixed entity, there being no singular standard for this concept. The Social construction of masculinities is also concerned with the traditional patterns of gender socialization, which result in producing masculinities according to societal norms [2].

Another theoretical approach that explores the connections between individuals’ sexuality and gender behaviours in a social context is called ‘Sexual Script Theory’. Based on sexual script theory, sexuality is shaped through experiences, and meanings are developed through social encounters within a historical period [38]. Gender scripts are culturally learned ways of thinking and behaving that men and women associate with to express their manliness and womanliness [39]. Scripts need to be analyzed from three dimensions: intra-psychic scenarios, interpersonal scenarios and cultural scenarios. This analysis provides an understanding of the process of script development amongst people, by deconstructing script development into reinforcement, modelling, rehearsal and symbolization [25].

In this study, both social construction of masculinities and sexual scripts theory provided a conceptual framework for exploring the masculine conceptualization among university men.

## Methods

Given the purpose of the study and its research questions, as well as the fact that the concept of masculinity is a phenomenon that has not been fully explored among Malaysian university men, we applied qualitative research as the methodological approach for collecting data. Qualitative research seeks to uncover meanings and to promote the understanding of the experiences of the research clients. This approach places primary value in interpreting the reasons for observable behaviours, as a way to help understand actions that are dynamic and social in foundation and structure [40,41]. Qualitative research methods have been successfully applied to many studies on sexuality and gender based issues among young people [5,7,10,13,35,42,43].

### Research setting and data collection

This study was carried out across universities based in Kuala Lumpur, the capital city of Malaysia. One of the research members, a psychologist and a member of the Students’ Association at a public university, acted as lead in organising the study sample. Being a skilled psychologist, he was trained during several meetings with the research team, and also participated in a qualitative research workshop to earn professional experience for this research. He developed a network of assistants from different universities, by inviting various people to participate in the study and distributing the research topic among the students involved.

A total of 34 undergraduate and postgraduate men participated in the study (Table 1). According to the theoretical assumption that socio-economic status and cultural background affect the meanings of masculinity among men [2], a purposeful sampling method was selected in order to capture a wide range of cultural diversity. Since Malaysia is a multi-ethnic society, the participants were selected from the country’s major races (Malay, Chinese and Indian). We also took into account several other factors, such as age (20–30 years old), status of the universities (public/private), levels of study (undergraduate/postgraduate) and fields of study. The criteria for inclusion were that participants had to be unmarried, heterosexual men.

**Table 1:** Characteristics of the interviewees (N = 34)

Age	Minimum: 20 yrs	Maximum: 30 yrs	Mean: 23.76 yrs	Median: 24 yrs	SD (±3.026 yrs)
Race	Chinese (N=13) 38%	Malay (N=11) 32%	Indian (N= 10) 29%		
Education Level	Undergraduate (N=21) 61%	Postgraduate (N=13) 39%			
University Status	Private (N=16) 47%	Public (N=18) 53%			
Religion	Muslim (N=11) 32%	Buddhist (N=10) 30%	Hindu (N=10) 30%	Christian (N=3) 8%	
Field of Study	Medical (N=6) 18%	Psychology (N=3) 9%	Engineering (N=7) 1%	Educational sciences (N=4) 12%	
	Art (N=2) 6%	Basic sciences (N=4) 12%	Business (N=4) 12%	Accounting (N=2) 6%	Law (N=2) 6%

A face to face interview technique consisting of open-ended questions was adopted to collect the data pertinent to this study. The interview protocol was prepared based on a literature review followed by a series of discussions among the research team. The interviews lasted between 45 and 110 minutes. They were conducted, according to the participants’ preferences, in the meeting/discussion/counselling rooms of the universities, with some even taking place in the university canteens.

### Interview guideline

• What comes to your mind when you hear the word masculinity?

• What characteristics define a man? (Probe for physical, emotional, social …)

• What is the role of a man in his life? (Probe for roles in society, family, romantic relationship, personal life…)

• How do you describe your social and personal lives? (Probe for life stories and daily activities, family life, romantic relationships…)

• How do you describe your goals and achievements in your life? (Probe for social life, family life, romantic relationships…)

There have been concerns regarding the interview language, which might affect the authenticity of the collected data [44,45]. Bahasa Malayu is the official language of Malaysia, with English being spoken as the secondary language, to the extent that most young people are fluent in English and use it when speaking to their peers. Since gender related concepts are highly affected by cultural and social norms, finding the right equivalent in any language to represent the full sense of the word used by participants in their cultural context and native language is an important issue [44].

To overcome this issue, the interviews were conducted using a combination of English and Bahasa Malayu by a native interviewer who was fluent in both of them. The language of each interview was chosen according to the interviewee’s preference. Out of 34 interviews, 21 interviews were conducted in English and 13 interviews in Bahasa Malayu. All of the interviews were conducted by a male interviewer, who made audio recordings of each session.

Prior to the study, ethics approval was obtained from the ethics committee of the University of Malaya, and written consent was obtained from all respondents. Respondents were made to fill out a confidential socio-demographic questionnaire before the interview, with data collection continuing until data saturation had occurred.

### Data management and analysis

All of the audio files were transcribed verbatim by the interviewer. The transcripts were sent off to the research clients so that the members could check over the transcribed data. The 13 transcripts that were conducted in Bahasa Malayu were translated to English by the same interviewer, and the translations were then sent to another reader (who is also fluent in both English and Bahasa Malayu) so as to ensure an accurate translation. Although it is not always possible to preserve the raw essence of certain sayings through translation, doing everything possible to maintain the message post translation was considered to be an important issue in terms of quality control for the sake of maintaining credibility regarding the translated data [46].

All text based data was edited and saved as text files to import into NVIVO Qualitative Data Management Software. All qualitative data was coded and analyzed using NVIVO-v8 software.

The process of data analysis ran alongside the process of data collection [47], and was carried out by the interviewer, the first author, and the main research investigator. A thematic analysis approach was used to extract meaningful themes from the transcripts. The first step was to identify, name, and explore the descriptive and conceptual components in the data. During this initial phase, the researchers went through several transcripts, reading them line by line to capture any meaningful concepts. Concepts and incidents that seemed to be related to the same phenomenon were grouped together under an assigned code. Then, related codes were categorized and merged into broader themes which corresponded to shared meanings of masculinity among university men [48].

To assure the credibility, transferability and dependability of the research, we adopted a combination of Creswell and Miller’s procedures and Maxwell’s ‘checklist of validity’ [49]. We presented the findings to the participants in a feedback session, where they were asked to comment on themes and interpretations that had emerged during the course of the study. Overall, they confirmed that the themes observed during the research accurately represented their understanding of masculinity.

We also presented the findings in seminars with our peers, and showed it to experts in qualitative research, through whom we obtained confirmation regarding our interpretation of the data. Some procedures that were suggested with the aim of improving the quality of future research included engaging in prolonged interaction with the research clients, collecting tick and rich data and looking for negative cases [49].

## Results

The findings of this study are presented under the heading Meanings of Masculinity (below), which depicts gender-related beliefs and attitudes among the participants. In this section, the research findings are discussed and supported with representative quotes from the interviews in detail.

### Meanings of masculinity

From the data collected several concepts emerged that reflected the meanings of masculinity from the participants’ view points. These included: “Having a good body shape”, “being respected”, “having success with women”, “being a family man” and “having financial independence”. Some of the concepts such as “having a good body shape” and “having success with women” were perceived as being the current requirements of masculinity, whereas “being a family man” and “having economic power” reflected future masculine roles as perceived by the participants.

*Having a good body shape* - the concept of having a fit and muscular body was a clear indicator of a manly appearance among the participants, which was associated with the notions of strength and power:

“*To me a true man has the traits of the real man*, *must be muscular*, *his body is muscular so he must be strong. He has to have body shape*, *like muscle*, *and he is supposed to do rough sports*, *so that*’*s a man to me*.” 23-year-old Malay

As the interviews continued, “ambiguity codes” were gradually incorporated into the concepts of masculinity. According to the respondents, having a muscular body is more about obtaining a pleasing atheistic appearance, in line with the body form favoured by fashion models and celebrities as seen in magazines and on television:

“*The new masculine men are more feminine men that you can see in people in the media like Justine Bieber* [a Canadian teen pop musician, singer and actor] *or the Korean boy bands. And*, *right now*, *nerds are actually becoming the new masculine man. They are more prone to appear on TV compared to macho men anymore*, *I like them as a role model*.” 24-year-old Malay

“*Basically*, *appearance*-*wise and all that*… *A muscular and good shape*, *like what you can see in fashion magazines*.” 29-year-old Indian

They also related a man’s body shape to the meaning of masculinity as a means through which they were able to attract women with the intention of forming heterosexual relationships, as one 24-year-old Malay man stated:

“*Masculinity means how to show your macho*-*ness*…*peacock style*… *a man presents himself by his body*, *his masculinity must be presented to attract women*… *a fit body shape attracts more*.”

Overall, the research clearly shows that young men’s perception of an ideal body image is changing from the traditional muscular man to a more aesthetic body, as heavily influenced by the media and the resulting life styles that become popular among young modern men.

*Being* (*becoming*) *a family man* - another theme emerged from the data which implied that some ideas of masculinity are tied to concepts such as familial responsibilities and leadership.

“*See in our lives*, *it all ends with a household. Meaning we will have a wife and kids. So the responsibility can be said to be 100%.But all that must be presented*… *Mental strength and how to manage your family*, *settling problems*…*so*, *he has to be intelligent. To me*, *all of this encapsulates*.” 26-year-old Malay

Attributes such as being a provider for the family (the bread winner), a hard worker, good leader (decisive), a problem solver, being knowledgeable and being a good father all correspond to this category. Although none of the participants were married, they clearly portrayed the future position of being a family man as one of their understandings of masculinity. They were often influenced by their religion and opinions gained from their parents’ roles, especially the father’s role. Muslim and Christian men more frequently referred to religious thoughts in their responses:

“*Normally in Christian Chinese families*, *man is the leader. So*, *when it comes to a man*, *if you are right*, *you make a decision. You are not dependent to anyone. You can stand on your own. You earn money and provide for family. I suppose a woman could have earned money for family too but*, *I assume that the breadwinner is more a masculine role*.” 23-year-old Chinese

‘Becoming a good father’ was a concept that seemed to be most heavily influenced by those with religious thoughts. As a Muslim man stated:

“*I think being a good father is very important*, *as Islam has encouraged us*, *we are responsible to raise righteous children. No matter how much you have earned in your life*, *if you are terrible in becoming a father*, *I think you basically fail*… *fail in terms of family*.” 26-year-old Malay

*Having success with women* - this theme was associated with the meanings of masculinity in the context of a heterosexual relationship. ‘Gentleman’ was the term most frequently used to portray a man with social grace, who as a result enjoys successful relationships with women.

“*You should balance both the macho man and the gentler side of the modern man. You should be polite and socialized. This is the social image of man that people accept these days*.” 26-year-Old Chinese

Having a “gentle character” and being able to maintain a “caring and communicative relationship” with women was also emphasized by the respondents.

“*Most girls don*’*t really think macho men are that cool anymore. They prefer the more slender*, *feminine men who are gentle and could listen to them and take care of them*.” 22-year-old Chinese

As a group of heterosexual men, the potential for relationships with women were central to their understanding of masculine identities. Success with women was considered as being a harmonic and respectful relationship with partners, by which the participants meant something more than a purely sexual arrangement. In this regard, they associated masculinity with a combination of stereotypical norms (such as taking initiative and mastering the romantic relationship) and non-stereotypical norms (such as being communicative, gentle and listening).

*Being respected* - according to the respondents, a man “must know how to carry himself in the public to earn the respect of the other men”. They linked the concept of “being respected” with “achievements” and “uniqueness” of a man in his life. As a 29-year-old Malay man stated:

“*When it comes to the guys*, *when they talk about their activity*, *they*’*ll be like congratulating them and all that*… *being proud of his level*…*he must think of a novel idea*, *he*’*ll be like wondering*, *being someone that supposed to be*… *it brings respect and confidence*, *meaning you stand up for yourself*.”

Participants tended to prioritize this concept over other meanings of masculinities, such as having a good body shape. As a 25-year-old Chinese man emphasized:

“*Respect is the most important thing if you ask about the meaning of masculinity to me. Just by having a good shape*, *but if you do not know how to talk to others*, *if you do not know how to carry yourself in the public*, *people don*’*t respect you*.”

Respondents linked the idea of being respected with their social status at the university. Being respected in terms of a university context was defined as having academic achievements, as well as being involved in campus activities and social events, especially when “taking leadership”.

“*I study very hard*, *and I try to take some responsibilities in the campus*, *for example*, *I was the president of cultural night ceremony last year and I am going to do it again*… *doing such things make you popular in the campus*, *people look at you as a leader*, *as a boss*… *they have respect for you*, *they say ohh look at this man*… *he is doing a big job*.” 22-year-old Malay

*Having financial independence* - respondents believed that “money matters in today’s life”, that it brings “power” and “confidence” to them. They mentioned that a “financially independent man” is more attractive to women, more capable of supporting his family and seen as being more focused on his life.

“*You know*, *becoming a confident man cannot be happened without having money. Of course*, *money is very important*… *to feel proud and independent*, *to focus on your goals and to enjoy your life*… *the higher income you have the more girls come around* [*laughing*].” 22-year-old Indian

As university students, they considered their fields of study as being an important factor in helping them to obtain the best paying jobs.

“*Engineering is one of the best paying jobs in Malaysia*, *that*’*s why I chose it. Here is a private university and I have to spend lots of money to graduate*… *it is like investment for my future*, *when I imagine myself as an engineer in a best paying company I feel assured and confident. It guarantees my future*.” 23-year-old Chinese

Medical students also mentioned their future career potential in terms of winning one of the better paying jobs, which would help secure their “economic power” and place in a “higher social class”. Universally, the participants believed that financial independence is an important marker of true masculinity.

## Discussion

This research was designed to explore the various meanings of masculinity among Malaysian university men. As a result, several core concepts arose that reflected the meanings of masculinity among the respondents. Social construction of masculinities recognizes the existence of multiple masculinities among men, and emphasizes the influence of social contexts in shaping the meanings of masculinities [3]. Socio-cultural factors, such as family environment, religion, public media and popular life style patterns help to shape and reinforce the meanings of masculinities among university men. As stated by Sexual Script Theory, meanings are developed through experiences, and gender scripts are learned in cultural contexts [25] so family, religious beliefs and social encounters all affect the respondents understanding of masculinities. These meanings were associated with a combination of traditional and non-traditional norms that generally benefit men who behave according to culturally dominant role expectations.

These findings could potentially provide a way for educators to help university men better express their masculinity in positive ways. For example, as obtaining financial independence after graduation is one of the main ideals of modern masculinity, educators should provide relevant counselling services to help students make more informed choices about their career paths and majors [24].

Our study also shows that men develop their own masculine hierarchy within the university social sphere, which puts more value on some members than the others, e.g. men with academic achievements or those who are involved in campus activities tend to feel more respected. Therefore, educators should provide services that aim to help under achieving students to improve themselves academically. They should also ensure to facilitate the involvement of students in campus events, in order to help individuals express their leadership qualities in a social environment so that they can distinguish themselves and earn respect.

By supporting the growth of masculinity within a university context it is possible to provide for a cross-cultural interaction among men who represent diverse backgrounds, beliefs and experiences. This can then lead to challenging their prejudgments and assumptions about masculinity, thus motivating them to form new, more positive meanings of masculinities [24,50].

In accordance with another study in Malaysia, which also reported the existence of multiple masculine identities among Malaysian men [35], our study shows that the respondents believe in a combination of stereotypical and non-stereotypical norms for how to act in the context of a heterosexual relationship. The rise of these non-traditional masculine norms could potentially result in role confusion [2]. As such, considering the centrality of a healthy heterosexual relationship in regards to the meanings of masculinity among respondents, educational programs and relationship counselling services should be provided on campus to help address any potential issues.

Similarly, a study of meanings of masculinity among American college men [24] showed that respondents associated the meanings of masculinity with being respected, being confident and self assured, assuming responsibility and embodying physical prowess. According to that study, respondents linked the meanings of masculinity with hyper masculine performances, such as alcohol consumption, objectification of women and pursuit of exclusively sexual relationships, which did not reflect the findings from our study.

An Asian study on important masculinity attributes demonstrated different perceptions of men in different countries [19]. While having a good job was considered as the most important masculine attribute in Malaysia, having lots of money, being in control, being seen as a man of honour and being a family man were considered as being the most important factors in China, Taiwan, Japan and Korea respectively. In contrast with our findings, Malaysian participants assigned the least importance to being physically attractive and having success with women. One reason for this result could be related to the older age composition of the study sample, as only 31 percent of the respondents belonged to the same age group as interviewed in our study (20–30 years old). So these findings may potentially demonstrate that different age groups have different perceptions, and as such the meanings of masculinity differ according to a person’s respective age group.

This study raises several questions that can be explored in future studies on experience of masculinity among university men. Studies could be designed regarding what masculinity concepts men learn from university life that distinguish them from other men in society. Another question is how upcoming life events such as marriage, becoming a father and developing a career affect men’s understanding of masculinity.

This study is not without its limitations. Despite the strategies we employed to establish trustworthiness, the interpretation and translation of the data might not fully represent the actual experiences of the participants. In addition, our sample was limited to university students in an urban environment, which is not representative of all young Malaysian men. A qualitative approach aims at exploring the theoretical patterns rather than generalizing, so if we were to try relate these findings to the general population, the composition of the sample would be of concern.

## Conclusions

This study reveals that the university context provides a particular culture that constructs and reinforces the meanings of masculinity among young men. Being aware of the existence of multiple meanings for masculinity challenges prejudgments and assumptions about the normative single-standard masculinity, which can motivate young men to form new opinions on the meanings of masculinity. University educators should provide services that facilitate the expression of all ranges of masculinities, so as to help construct healthy masculinities to enable university men to behave according to their role preferences beyond the pressure of acting in accordance with normative expectations.

## Competing interests

The authors declare that they have no competing interests.

## Authors’ contributions

All the authors contributed equally in the research process. All authors read and approved the final manuscript.

## Pre-publication history

The pre-publication history for this paper can be accessed here:

http://www.biomedcentral.com/1471-2458/13/1062/prepub
